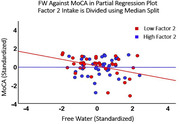# Healthy Dietary Intake Diminishes the Effect of Vascular Pathology on Cognitive Performance in Older Adults

**DOI:** 10.1002/alz.091804

**Published:** 2025-01-09

**Authors:** Christopher E. Bauer, Valentinos Zachariou, Colleen Pappas, Pauline Maillard, Arvind Caprihan, Claudia L Satizabal, Brian T Gold

**Affiliations:** ^1^ University of Kentucky, Lexington, KY USA; ^2^ Alzheimer's Disease Research Center, University of California Davis, Sacramento, CA USA; ^3^ The Mind Research Network, Albuquerque, NM USA; ^4^ Glenn Biggs Institute for Alzheimer’s & Neurodegenerative Diseases, University of Texas Health Science Center at San Antonio, San Antonio, TX USA

## Abstract

**Background:**

Cognitive reserve (CR) in the context of Alzheimer’s’ disease has been widely studied, yet less is known about how CR protects against vascular brain pathologies. Here, we explored whether dietary factors might attenuate the association between magnetic resonance imaging (MRI)‐derived vascular biomarkers and cognition.

**Method:**

Seventy‐one older adults (ages 60‐85) were scanned using a 3‐Tesla MRI Siemens Magnetom Prisma at the University of Kentucky. Data from a 3D T1‐weighted sequence, a 3D fluid‐attenuated inversion recovery sequence, and a 126‐direction diffusion MRI sequence were acquired. The vascular biomarkers used [White Matter Hyperintensity Volume (WMHV), Free Water (FW), and Peak Width of Skeletonized Mean Diffusivity (PSMD)] were developed and validated through the MarkVCID consortium (https://markvcid.partners.org). WMHV was computed using a 4‐tissue segmentation model, mean FW in all white matter was calculated using a two‐compartment model, and PSMD was calculated as the difference between the 95^th^ and 5^th^ percentiles in white matter MD values. Grey matter volume (GMV; non‐vascular biomarker) and intracranial volume (ICV) were estimated using FreeSurfer. The “Newly Developed Antioxidant Nutrient Questionnaire” was used to quantify dietary‐intake for the preceding month. Nutrients were grouped into nutrition factors based on previous literature and factor analysis (Factor 1= representing fruits and vegetables; Factor 2= representing nuts, healthy oils, and fish; Factor 3= representing green tea). All participants completed the Montreal Cognitive Assessment (MoCA). Multivariate linear regression models tested whether dietary factors, vascular biomarkers, and/or their interaction (i.e. moderation) were associated with MoCA scores. All models controlled for age, sex, ICV, and education.

**Result:**

There were no significant main effects of WMHV, FW, PSMD, or GMV on MoCA scores. However, Factor 2 (but not other factors) positively moderated all 3 vascular biomarkers [WMHV (β=0.309, p=0.009); FW (β=0.324, p=0.007); PSMD (β=0.354, p=0.008)] such that a negative association between vascular markers and MoCA scores was only present in those with low but not high Factor 2 intake (Figure 1). Factor 2 did not moderate the association between non‐vascular biomarkers (i.e. GMV) and MoCA scores.

**Conclusion:**

Our results suggest that consuming more nuts, healthy oils, and fish may help protect against vascular contributions to cognitive impairment.